# (*Z*,*E*,*Z*)-1,6-Di-1-naphthyl­hexa-1,3,5-triene

**DOI:** 10.1107/S1600536809000592

**Published:** 2009-01-14

**Authors:** Yoriko Sonoda, Masaru Yoshida, Midori Goto

**Affiliations:** a Nanotechnology Research Institute, National Institute of Advanced Industrial Science and Technology (AIST), 1-1-1 Higashi, Tsukuba, Ibaraki 305-8565, Japan; b Technical Center, AIST, 1-1-1 Higashi, Tsukuba, Ibaraki 305-8565, Japan

## Abstract

The title compound, C_26_H_20_, lies about an inversion centre. The naphthalene unit and the hexa­triene chain are each approximately planar (maximum deviations of 0.0143 and 0.0042 Å, respectively), and are inclined to one another at a dihedral angle of 49.20 (4)°. The dihedral angle between the two naphthalene ring systems of neighboring mol­ecules is 85.71 (4)°.

## Related literature

For the potential use of α,ω-diaryl­polyenes as non-linear optical materials, see: Geskin *et al.* (2003 ▶); Rumi *et al.* (2000 ▶). For a study of the relationship between the crystal structure and the photophysical properties of 1,6-diaryl­hexa-1,3,5-trienes, see: Sonoda *et al.* (2006 ▶); Sonoda, Goto *et al*. (2007 ▶). For related structures, see: Aldoshin *et al.* (1984 ▶); Sonoda *et al.* (2005 ▶); Sonoda, Tsuzuki *et al*. (2007 ▶).
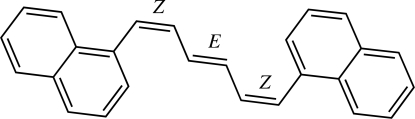


## Experimental

### 

#### Crystal data

C_26_H_20_
*M* *_r_* = 332.42
Monoclinic, 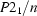

*a* = 5.0071 (8) Å
*b* = 11.0709 (17) Å
*c* = 16.110 (3) Å
β = 96.535 (3)°
*V* = 887.2 (3) Å^3^
*Z* = 2
Mo *K*α radiation
μ = 0.07 mm^−1^
*T* = 203 (2) K
0.30 × 0.10 × 0.05 mm

#### Data collection

Bruker SMART CCD area-detector diffractometer
Absorption correction: multi-scan (*SADABS*; Sheldrick, 1996 ▶) *T*_min_ = 0.910, *T*_max_ = 0.997
5367 measured reflections
2023 independent reflections
1366 reflections with *I* > 2σ(*I*)
*R*_int_ = 0.027

#### Refinement

*R*[*F* ^2^ > 2σ(*F* ^2^)] = 0.043
*wR*(*F* ^2^) = 0.114
*S* = 1.01
2023 reflections
118 parameters
H-atom parameters constrained
Δρ_max_ = 0.16 e Å^−3^
Δρ_min_ = −0.16 e Å^−3^

### 

Data collection: *SMART* (Bruker, 2001 ▶); cell refinement: *SAINT* (Bruker, 2001 ▶); data reduction: *SAINT*; program(s) used to solve structure: *SHELXTL* (Sheldrick, 2008 ▶); program(s) used to refine structure: *SHELXTL*; molecular graphics: *SHELXTL*; software used to prepare material for publication: *SHELXTL*.

## Supplementary Material

Crystal structure: contains datablocks I, global. DOI: 10.1107/S1600536809000592/at2699sup1.cif

Structure factors: contains datablocks I. DOI: 10.1107/S1600536809000592/at2699Isup2.hkl

Additional supplementary materials:  crystallographic information; 3D view; checkCIF report

## Figures and Tables

**Fig. 1.**
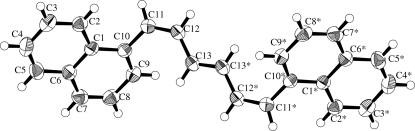
A view of the molecular structure and the atom-numbering scheme of (I). Displacement ellipsoids are drawn at the 50% probability level and H atoms are shown as small spheres of arbitrary radii. The title compound lies about an inversion centre [(*) -x, -y, -z].

**Fig. 2.**
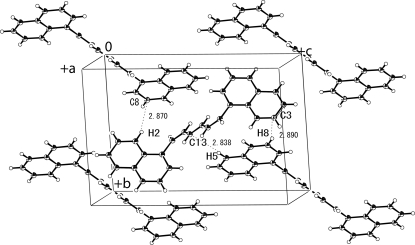
A packing diagram of (I) illustrating intermolecular contacts associated with hydrogen atoms H2, H5 and H8 of the naphthalene ring.

## supplementary crystallographic information

### Comment

α,ω-Diarylpolyenes are known as fluorescent molecules in solution, and are also attractive because of their potential use as non-linear optical materials (Rumi *et al.*, 2000; Geskin *et al.*, 2003). During an ongoing study on the relationship between the crystal structure and the photophysical properties of 1,6-diarylhexa-1,3,5-trienes (Sonoda *et al.*, 2006; Sonoda, Goto *et al.*, 2007), we obtained the title compound (I), whose structure we report here.

In the present compound, the averaged value of the C—C single bond length in the hexatriene chain is 1.457 Å, that of the C=C double bond length is 1.341 Å, and the resulting bond-length alternation (δr, the difference between the single and double bond lengths) is 0.116 Å. The title compound lies about an inversion centre.

The naphthalene ring and the hexatriene chain are approximately planar, with the maximum deviations of 0.0143 and 0.0042 Å from the least-squares planes, respectively (Fig. 1). The dihedral angle between the ring and the chain is 49.20 (4)°. Thus, the steric hindrance between C9—H and C13—H is minimized by the twisting around the C10—C11 single bond. C—C—C internal bond angles in the hexatriene chain are all somewhat wider than 120°, which also minimizes the steric hindrance.

The structure of (I) can be compared with those of (*Z*,*E*,*Z*)-1,6-diphenylhexa-1,3,5-triene 4,4'-dicarboxylic acid dialkyl esters (Sonoda *et al.*, 2005). In the case of the dimethyl ester, for example, δr is 0.111 Å and other geometrical parameters for the triene chain including C—C—C bond angles are all comparable with the values in (I). Also in this compound, the benzene ring and the triene chain are nearly planar for conjugation. The torsion angle of the single bond between the ring and the chain is 41.0 (2)°, significantly smaller than the C9—C10—C11—C12 angle in (I). This is probably due to the additional steric hindrance between C2—H and C11—H in (I).

For another related structure of (*Z*)-1,2-di(1-naphthyl)ethylene, the twisting not only around the naphthalene-ethylene single bond but also around the C=C double bond minimize the large steric hindrance between the two hydrogen atoms at the 2-position of the naphthalene ring (Aldoshin *et al.*, 1984). Different from the high planarity of the hexatriene unit in (I), the C—C═C—C torsion angle in this compound is 14.6°. While, the torsion angle of 44.1° about the naphthalene-ethylene single bond is similar to or even slightly smaller than the corresponding angle in (I).

In the crystal structure of (I), there are some C—H···π contacts (Fig. 2). The dihedral angle between the two naphthalene rings of the neighboring molecules is 85.71 (4)°.

### Experimental

Compound (I) was synthesized by the Wittig reaction of 1-naphthaldehyde and (*E*)-but-2-ene-1,4-bis(triphenylphosphonium chloride). The reaction gave a mixture of *Z*,*E*,*Z* and E,*E*,*E* isomers (predominantly *Z*,*E*,*Z*), from which the *Z*,*E*,*Z* isomer (I) was crystallized from dichloromethane by slow evaporation at room temperature in the dark. ^1^H NMR (CDCl_3_, 300 MHz): δ 7.95–7.99 (2*H*, m, arom.), 7.80–7.90 (4*H*, m, arom.), 7.43–7.55 (8*H*, m, arom.), 6.95 (2*H*, d, J = 11.1 Hz, triene), 6.72 (2*H*, dd, J = 7.7, 3.0 Hz, triene), 6.47 (2*H*, ddd, J = 11.0, 7.8, 3.2 Hz, triene).

### Refinement

All non-hydrogen atoms were refined anisotropically and hydrogen atoms were located by geometric considerations and refined as riding on their carrier atoms [ C—H = 0.94 Å, U_eq_ = 1.2 U_iso_(C) ].

### Crystal data

**Table:**

C_26_H_20_	*F*(000) = 352
*M**_r_* = 332.42	*D*_x_ = 1.244 Mg m^−^^3^
Monoclinic, *P*2_1_/*n*	Mo *K*α radiation, λ = 0.71073 Å
Hall symbol: -P 2yn	Cell parameters from 1467 reflections
*a* = 5.0071 (8) Å	θ = 2.6–27.1°
*b* = 11.0709 (17) Å	µ = 0.07 mm^−^^1^
*c* = 16.110 (3) Å	*T* = 203 K
β = 96.535 (3)°	Rectangular, pale yellow
*V* = 887.2 (3) Å^3^	0.30 × 0.10 × 0.05 mm
*Z* = 2	

### Data collection

**Table:**

Bruker SMART CCD area-detector diffractometer	2023 independent reflections
Radiation source: rotating unit	1366 reflections with *I* > 2σ(*I*)
graphite	*R*_int_ = 0.027
Detector resolution: 8.366 pixels mm^-1^	θ_max_ = 28.3°, θ_min_ = 2.2°
φ and ω scans	*h* = −6→6
Absorption correction: multi-scan (*SADABS*; Sheldrick, 1996)	*k* = −6→14
*T*_min_ = 0.910, *T*_max_ = 0.997	*l* = −21→20
5367 measured reflections	

### Refinement

**Table:**

Refinement on *F*^2^	Primary atom site location: structure-invariant direct methods
Least-squares matrix: full	Secondary atom site location: difference Fourier map
*R*[*F*^2^ > 2σ(*F*^2^)] = 0.043	Hydrogen site location: inferred from neighbouring sites
*wR*(*F*^2^) = 0.114	H-atom parameters constrained
*S* = 1.00	*w* = 1/[σ^2^(*F*_o_^2^) + (0.0527*P*)^2^ + 0.1208*P*] where *P* = (*F*_o_^2^ + 2*F*_c_^2^)/3
2023 reflections	(Δ/σ)_max_ = 0.001
118 parameters	Δρ_max_ = 0.16 e Å^−^^3^
0 restraints	Δρ_min_ = −0.16 e Å^−^^3^

### Special details

**Table:**

Experimental. Sheldrick, G. *M*. (1996). *SADABS*, program for scaling and correction of area detector data. University of Göttingen, Germany.
Geometry. All e.s.d.'s (except the e.s.d. in the dihedral angle between two l.s. planes) are estimated using the full covariance matrix. The cell e.s.d.'s are taken into account individually in the estimation of e.s.d.'s in distances, angles and torsion angles; correlations between e.s.d.'s in cell parameters are only used when they are defined by crystal symmetry. An approximate (isotropic) treatment of cell e.s.d.'s is used for estimating e.s.d.'s involving l.s. planes.
Refinement. Refinement of *F*^2^ against ALL reflections. The weighted *R*-factor *wR* and goodness of fit *S* are based on *F*^2^, conventional *R*-factors *R* are based on *F*, with *F* set to zero for negative *F*^2^. The threshold expression of *F*^2^ > σ(*F*^2^) is used only for calculating *R*-factors(gt) *etc*. and is not relevant to the choice of reflections for refinement. *R*-factors based on *F*^2^ are statistically about twice as large as those based on *F*, and *R*- factors based on ALL data will be even larger.

### Fractional atomic coordinates and isotropic or equivalent isotropic displacement parameters (Å^2^)

**Table:**

	*x*	*y*	*z*	*U*_iso_*/*U*_eq_	
C1	0.0718 (2)	0.18611 (12)	0.28101 (8)	0.0349 (3)	
C2	0.2135 (3)	0.10461 (14)	0.33764 (9)	0.0425 (4)	
H2	0.3383	0.0511	0.3183	0.051*	
C3	0.1719 (3)	0.10250 (15)	0.42018 (9)	0.0506 (4)	
H3	0.2711	0.0489	0.4570	0.061*	
C4	−0.0171 (3)	0.17947 (16)	0.45017 (9)	0.0536 (4)	
H4	−0.0446	0.1773	0.5069	0.064*	
C5	−0.1600 (3)	0.25694 (15)	0.39761 (9)	0.0501 (4)	
H5	−0.2894	0.3068	0.4182	0.060*	
C6	−0.1188 (3)	0.26465 (13)	0.31206 (8)	0.0399 (3)	
C7	−0.2601 (3)	0.34839 (14)	0.25750 (10)	0.0476 (4)	
H7	−0.3870	0.4002	0.2776	0.057*	
C8	−0.2140 (3)	0.35465 (14)	0.17611 (10)	0.0482 (4)	
H8	−0.3063	0.4120	0.1406	0.058*	
C9	−0.0295 (3)	0.27621 (13)	0.14452 (9)	0.0428 (4)	
H9	−0.0016	0.2818	0.0879	0.051*	
C10	0.1111 (3)	0.19160 (13)	0.19424 (8)	0.0369 (3)	
C11	0.3027 (3)	0.10910 (13)	0.16049 (8)	0.0411 (3)	
H11	0.4733	0.1020	0.1911	0.049*	
C12	0.2587 (3)	0.04314 (13)	0.09057 (8)	0.0407 (3)	
H12	0.4052	−0.0023	0.0761	0.049*	
C13	0.0132 (3)	0.03363 (13)	0.03492 (8)	0.0386 (3)	
H13	−0.1374	0.0771	0.0483	0.046*	

### Atomic displacement parameters (Å^2^)

**Table:**

	*U*^11^	*U*^22^	*U*^33^	*U*^12^	*U*^13^	*U*^23^
C1	0.0329 (6)	0.0326 (7)	0.0385 (7)	−0.0064 (6)	0.0006 (5)	−0.0059 (6)
C2	0.0416 (7)	0.0409 (8)	0.0441 (8)	−0.0017 (7)	0.0005 (6)	−0.0029 (7)
C3	0.0553 (9)	0.0523 (10)	0.0422 (8)	−0.0077 (8)	−0.0030 (7)	0.0048 (7)
C4	0.0622 (10)	0.0612 (11)	0.0381 (8)	−0.0137 (9)	0.0089 (7)	−0.0070 (8)
C5	0.0512 (9)	0.0513 (10)	0.0492 (9)	−0.0072 (8)	0.0121 (7)	−0.0165 (8)
C6	0.0383 (7)	0.0377 (8)	0.0435 (8)	−0.0067 (6)	0.0034 (6)	−0.0116 (6)
C7	0.0439 (8)	0.0402 (8)	0.0579 (9)	0.0055 (7)	0.0028 (7)	−0.0130 (7)
C8	0.0502 (8)	0.0384 (8)	0.0533 (9)	0.0053 (7)	−0.0059 (7)	−0.0020 (7)
C9	0.0464 (8)	0.0419 (8)	0.0394 (7)	−0.0024 (7)	0.0017 (6)	−0.0013 (6)
C10	0.0346 (7)	0.0366 (8)	0.0392 (7)	−0.0057 (6)	0.0023 (5)	−0.0051 (6)
C11	0.0360 (7)	0.0463 (9)	0.0409 (7)	−0.0001 (6)	0.0042 (6)	−0.0021 (7)
C12	0.0383 (7)	0.0434 (8)	0.0419 (7)	0.0007 (6)	0.0110 (6)	−0.0015 (7)
C13	0.0388 (7)	0.0384 (8)	0.0404 (7)	−0.0014 (6)	0.0127 (6)	0.0004 (6)

### Geometric parameters (Å, °)

**Table:**

C1—C2	1.4153 (19)	C7—H7	0.9400
C1—C6	1.4232 (19)	C8—C9	1.405 (2)
C1—C10	1.4350 (18)	C8—H8	0.9400
C2—C3	1.369 (2)	C9—C10	1.3736 (19)
C2—H2	0.9400	C9—H9	0.9400
C3—C4	1.400 (2)	C10—C11	1.4727 (19)
C3—H3	0.9400	C11—C12	1.3395 (19)
C4—C5	1.351 (2)	C11—H11	0.9400
C4—H4	0.9400	C12—C13	1.4404 (18)
C5—C6	1.419 (2)	C12—H12	0.9400
C5—H5	0.9400	C13—C13^i^	1.343 (3)
C6—C7	1.411 (2)	C13—H13	0.9400
C7—C8	1.359 (2)		
			
C2—C1—C6	118.04 (13)	C6—C7—H7	119.8
C2—C1—C10	122.72 (13)	C7—C8—C9	120.61 (14)
C6—C1—C10	119.24 (12)	C7—C8—H8	119.7
C3—C2—C1	121.08 (14)	C9—C8—H8	119.7
C3—C2—H2	119.5	C10—C9—C8	121.66 (13)
C1—C2—H2	119.5	C10—C9—H9	119.2
C2—C3—C4	120.56 (15)	C8—C9—H9	119.2
C2—C3—H3	119.7	C9—C10—C1	118.61 (13)
C4—C3—H3	119.7	C9—C10—C11	121.32 (13)
C5—C4—C3	120.03 (14)	C1—C10—C11	120.06 (12)
C5—C4—H4	120.0	C12—C11—C10	126.65 (12)
C3—C4—H4	120.0	C12—C11—H11	116.7
C4—C5—C6	121.45 (15)	C10—C11—H11	116.7
C4—C5—H5	119.3	C11—C12—C13	127.61 (13)
C6—C5—H5	119.3	C11—C12—H12	116.2
C7—C6—C5	121.78 (14)	C13—C12—H12	116.2
C7—C6—C1	119.41 (13)	C13^i^—C13—C12	123.89 (16)
C5—C6—C1	118.81 (14)	C13^i^—C13—H13	118.1
C8—C7—C6	120.41 (14)	C12—C13—H13	118.1
C8—C7—H7	119.8		
			
C6—C1—C2—C3	−1.0 (2)	C6—C7—C8—C9	1.4 (2)
C10—C1—C2—C3	179.57 (13)	C7—C8—C9—C10	−0.4 (2)
C1—C2—C3—C4	1.4 (2)	C8—C9—C10—C1	−1.6 (2)
C2—C3—C4—C5	−0.1 (2)	C8—C9—C10—C11	179.55 (13)
C3—C4—C5—C6	−1.5 (2)	C2—C1—C10—C9	−178.01 (13)
C4—C5—C6—C7	−177.64 (14)	C6—C1—C10—C9	2.54 (18)
C4—C5—C6—C1	1.9 (2)	C2—C1—C10—C11	0.90 (19)
C2—C1—C6—C7	178.92 (12)	C6—C1—C10—C11	−178.55 (12)
C10—C1—C6—C7	−1.60 (19)	C9—C10—C11—C12	−48.6 (2)
C2—C1—C6—C5	−0.66 (18)	C1—C10—C11—C12	132.53 (15)
C10—C1—C6—C5	178.82 (12)	C10—C11—C12—C13	−3.0 (2)
C5—C6—C7—C8	179.19 (14)	C11—C12—C13—C13^i^	179.04 (17)
C1—C6—C7—C8	−0.4 (2)		

Symmetry codes: (i) −*x*, −*y*, −*z*.
